# Supplementary material for: Topological scoring of protein interaction networks
Source: Nat Commun. 2019 Mar 8;10:1118. doi: 10.1038/s41467-019-09123-y (PMC6408525; doi:10.1038/s41467-019-09123-y)
Supplement: Supplementary file 1 — Supplementary Information [file 41467_2019_9123_MOESM1_ESM.pdf]

# Supplementary Information

## Topological Scoring of Protein Interaction Networks

M.E. Sardiu *et al.*

## Comparison of TopS to Alternative Pipelines

### Supplementary Methods

The accuracy of TopS was compared to previously published methods for scoring protein interactions: CompPASS [1], QSPEC [2], and SAINT [3]. The data was processed using standard workflows as suggested by the authors. QSPEC [2] analyses were performed online through the web server (http://www.nesvilab.org/qspec.php/), while SAINT [3] was downloaded from sourceforge using SAINTexpress-v3.6.3-2018 with default parameters for rapid and robust scoring of datasets. For QSPEC [2], baits, preys, protein length, and spectral counts were used as input and the normalization option was selected. The SAINT analysis [3] was performed using spectral counts and returned SAINT score and Bayesian false discovery rate-BFDR. For the CompPASS [1] method, we used the Z-score and WD-score (i.e. as suggested by the authors in the original paper) computed by the shiny app at http://bioplex.hms.harvard.edu/comppass/. In contrast to TopS, both SAINT [3] and CompPASS [1] required a formatted file as an input, with columns labeled as experiment.id, replicate, experiment type, bait, prey, and spectral count.

The performance of the three different pipelines for scoring protein interaction was evaluated using the three datasets described in the main text: the human DNA repair (17 baits), yeast INO80 (14 baits), and yeast SWI/SNF (10 baits) datasets (Supplementary Data 8-10). The methods were also assessed on a recently published dataset of the human polycomb complexome consisting of 174 APMS measurements, 64 baits, and 9,853 candidate interactions (Supplementary Data 11). In all, a total of 105 APMS runs were analyzed by all three of the scoring pipelines.

To appropriately compare TopS results with QSPEC [2] and SAINT [3] results, every bait was compared against all other baits in the dataset, which were considered positive controls. For

example, in the INO80 dataset, if IES1 was the bait, the remaining baits in the dataset (i.e. IES3, IES5, IES2, IES6, INO80, RVB1, RVB2, ARP5, ARP8, ARP4, TAF14, IES4 and NHP10) were considered positive controls. Thus, the number of scoring algorithms computed for a dataset is equal to the number of baits in the dataset. A total of 28 binary comparisons (one bait vs all others) were hence performed for the INO80 dataset, 20 comparisons for the SWI/SNF dataset and, 34 comparisons for the DNA repair dataset using QSPEC [2] or SAINT [3] computational algorithms. Since CompPASS [1] does not required a definition for negative controls, all controls were treated the same as any other bait in the dataset, similarly to the TopS approach. A total of 85 scoring algorithms using QSPEC [2], SAINT [3], and CompPASS were hence computed for the three datasets. We considered high scoring interactions for QSPEC [2] and SAINT [3]in the region Z-scores above 2 and a SAINT score above 0.5 and BFDR less than 0.05. In the case of CompPASS [1], interactions that scored greater than 1.5 Z- or WD-scores were considered significant.

**Supplementary Discussion**

The standard approach in the field of analysis of protein complexes is to compare bait affinity purifications to negative control purifications. CompPASS [1], SAINT [3], and TopS all are capable of fully determining components of protein complexes when bait affinity purifications are compared to negative control datasets (Supplemental Figure 10A). All three methods performed well by recovering all 15 subunits of the INO80 and all 12 subunits of SWI/SNF with high probability by SAINT or high scores by CompPASS and TopS (Supplementary Figure 10A). Next, the accuracy of each scoring method on potential direct protein interactions was evaluated by its recall rate for the set of 75 well-characterized interactions from crosslinking data for the INO80 [5] and SWI/SNF [6] complexes. In the case of the DNA repair dataset, we

benchmarked the high TopS interactions (i.e. TopS ≥20) against the BioGRID database [7] and we calculated the recall for the 121 selected interactions (Supplementary Data 8 and Supplementary Figure 9). At a threshold TopS score of 20, a total of 130 (including 9 baits with themselves) interactions overlapped with the BioGRID database [7] and 669 interactions overlapped with a score of 10.

The TopS score was the most accurate among all the tested scores. For example, for the INO80 complex, 27 direct interactions were reported from crosslinking experiments [5], and the recall number of interactions for CompPASS [1], SAINT [3], and TopS scores was 8, 11, and 21 high confidence interactions, respectively. In the case of the SWI/SNF complex, 48 direct interactions were detected by crosslinking, and the recall for CompPASS [1], SAINT [3], and TopS scores was 14, 1, and 30 high confidence interactions, respectively (Supplementary Data 9 and 10). For the DNA repair dataset, at the threshold TopS score of 20, SAINT [3] selected 34 (31%) interactions, while CompPASS [1] selected 88 (73%) interactions out of 121 BioGRID interactions (Supplementary Data 8). Overall, in all three cases with respect to recall of known interactions, TopS outperformed CompPASS [1] and SAINT [3] (Supplementary Figure 10A).

CompPASS [1] and SAINT [3] performed better for the larger DNA repair dataset than on the smaller yeast datasets (Supplementary Figure 10B). For example, the analysis on the DNA repair dataset with TopS resulted in the selection of 998 interaction pairs with a TopS score above 20 (see Supplementary Data 8 for details of the analysis). At this cutoff, 787 (79%) proteins were overlapping with CompPASS [1] result with a Z or WD score greater than 1.5, resulting in 211 additional proteins detected by TopS (Supplementary Data 8). CompPASS [1] detected 884 proteins, among which 787 (89%) were also detected by TopS with a cutoff of 20.

However, with a more flexible cutoff of TopS ≥10, only a 35 proteins discrepancy was observed, which implies that 96% of the proteins detected by CompPASS [1] were recovered by TopS.

By inspecting this difference, we found that TopS assigned high scores to proteins that were previously reported to interact with the bait, whereas CompPASS [1] failed to identify some of these connections. For example, the association between WDR76 and the TC- complex was discussed by Gallina *et al.* [8]. BioGRID [7] also reported several associations between CBX proteins and zinc finger domains, which were totally captured by TopS but not all by CompPASS [1]. MSH2 has the lowest overlap between the two methods, with TopS recovering reported interactions missed by CompPASS, such as the association between eIF3 complex and MSH2 (Figure 3). Evidence from BioGRID [7] confirms that the EIF3B subunit of the eIF3 complex associates with MSH2, however CompPASS [1] assigned a negative score to this interaction. QSPEC [2] produced similar results as TopS in terms of Z-scores, however, the calculated FDR was not very informative especially for the smaller datasets due to the presence of zeros values in the APMS analyses (Supplementary Figures 1 and 5). In spite of these comparisons of TopS to current pipelines, it is important to note that TopS is a complimentary approach. TopS is likely best used in addition to these pipelines to provide a different interpretation of a dataset with a particular focus on extreme TopS values across a dataset.

## Supplementary References


1. Sowa, M. E., Bennett, E. J., Gygi, S. P., and Harper, J. W., Defining the human deubiquitinating enzyme interaction landscape. *Cell* **138**, 389-403 (2009).
2. Choi, H., Fermin, D., and Nesvizhskii, A. I., Significance analysis of spectral count data in label-free shotgun proteomics. *Mol. Cell. Proteomics* **7**, 2373-2385 (2008).
3. Choi, H. *et al.*, SAINT: probabilistic scoring of affinity purification-mass spectrometry data. *Nat. Methods* **8**, 70-73 (2011).
4. Mellacheruvu, D. *et al.*, The CRAPome: a contaminant repository for affinity purification-mass spectrometry data. *Nat. Methods* **10**, 730-736 (2013).

5. Tosi, A. *et al.*, Structure and subunit topology of the INO80 chromatin remodeler and its nucleosome complex. *Cell* **154**, 1207-1219 (2013).
6. Sen, P. *et al.*, Loss of Snf5 Induces Formation of an Aberrant SWI/SNF Complex. *Cell Rep.* **18**, 2135-2147 (2017).
7. Chatr-Aryamontri, A. *et al.*, The BioGRID interaction database: 2015 update. *Nucleic Acids Res.* **43**, D470-478 (2015).
8. Gallina, I. *et al.*, Cmr1/WDR76 defines a nuclear genotoxic stress body linking genome integrity and protein quality control. *Nat. Commun.* **6**, 6533 (2015).

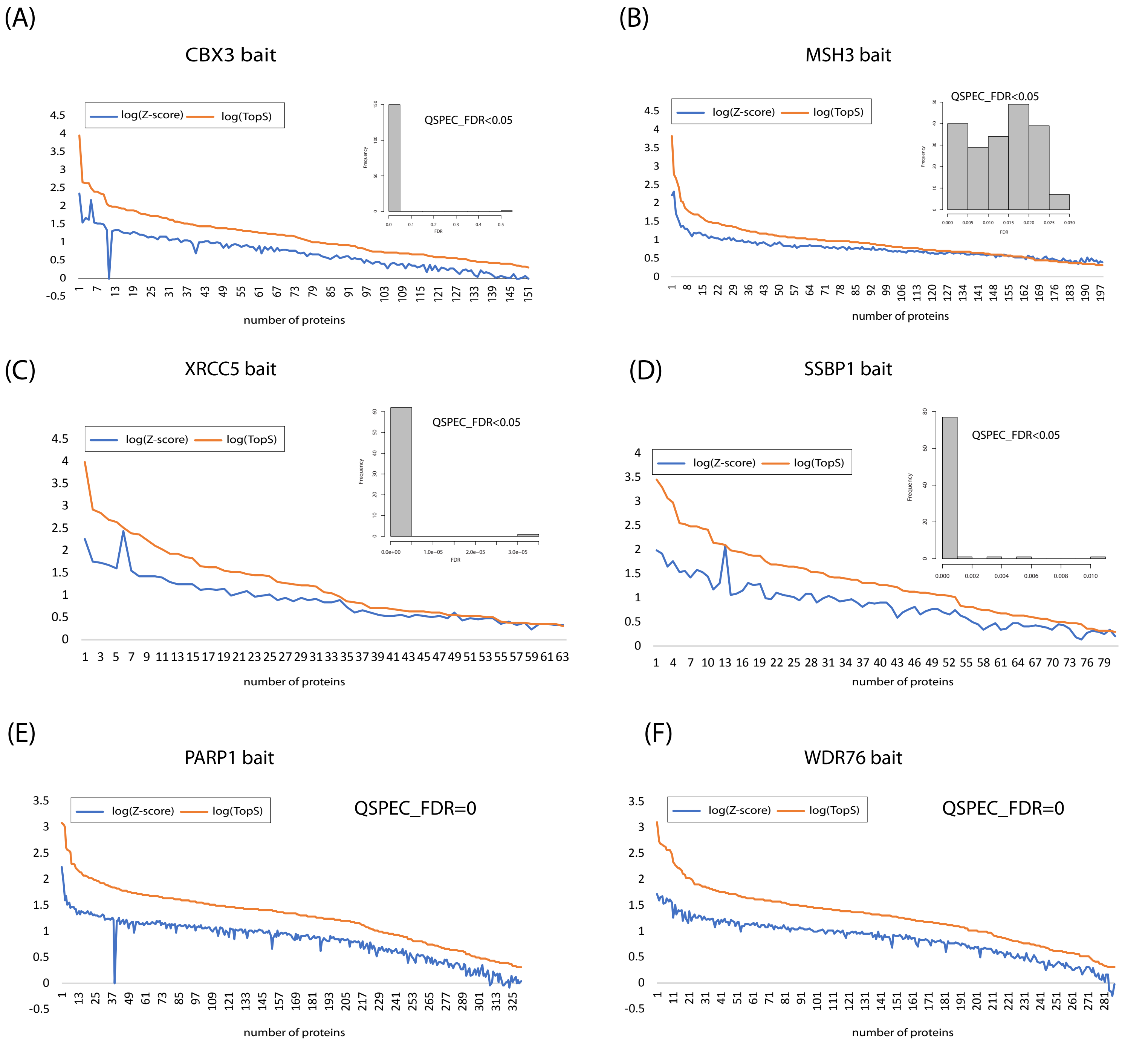


**Supplementary Figure 1. Distribution of the TopS, Z-scores and FDR values from QSPEC for the baits in the DNA repair dataset.** Number of proteins within a TopS threshold greater than 2 versus logarithm of TopS and Z-scores in the following baits: (A) CBX3, (B) MSH3, (C) XRCC5, (D) SSBP1, (E) PARP1 and (F) WDR76.

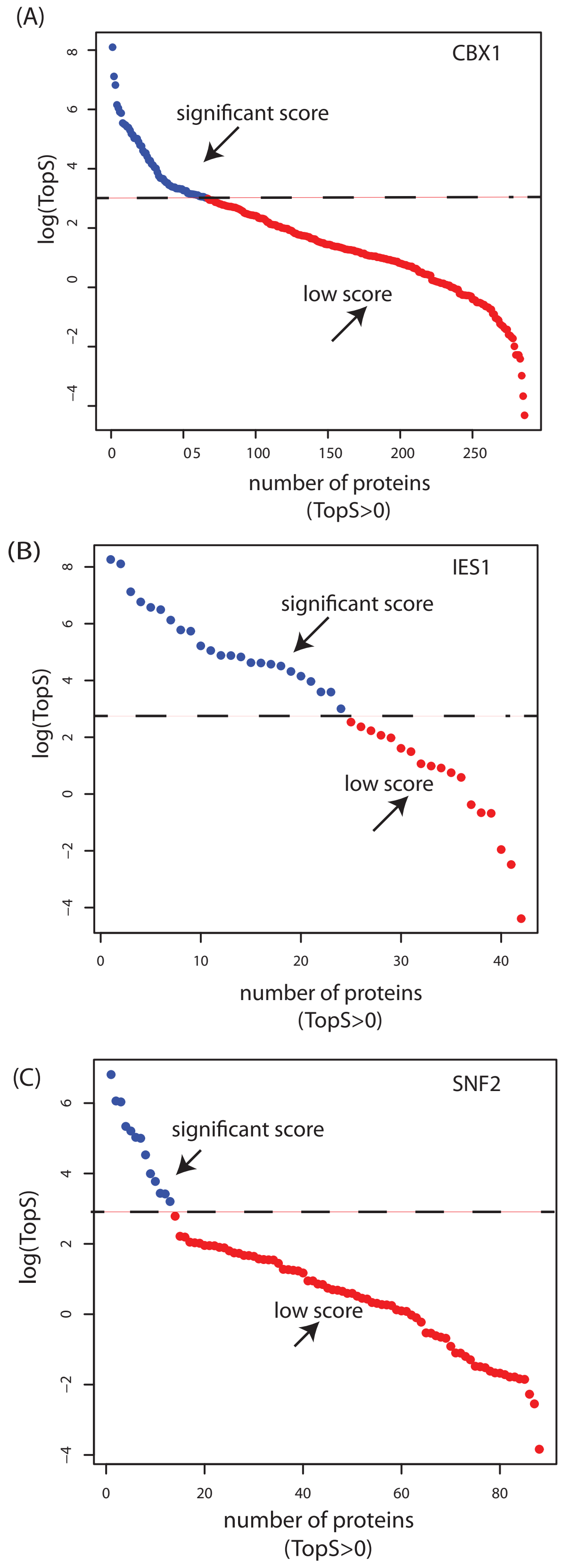


**Supplementary Figure 2. Distribution of the topological scores.** TopS calculated for prey proteins in the (A) CBX1, (B) IES1, and (C) SNF2 baits are represented as a linear log distribution. Proteins with TopS greater than 20 and lower than 20 are in blue and red, respectively.

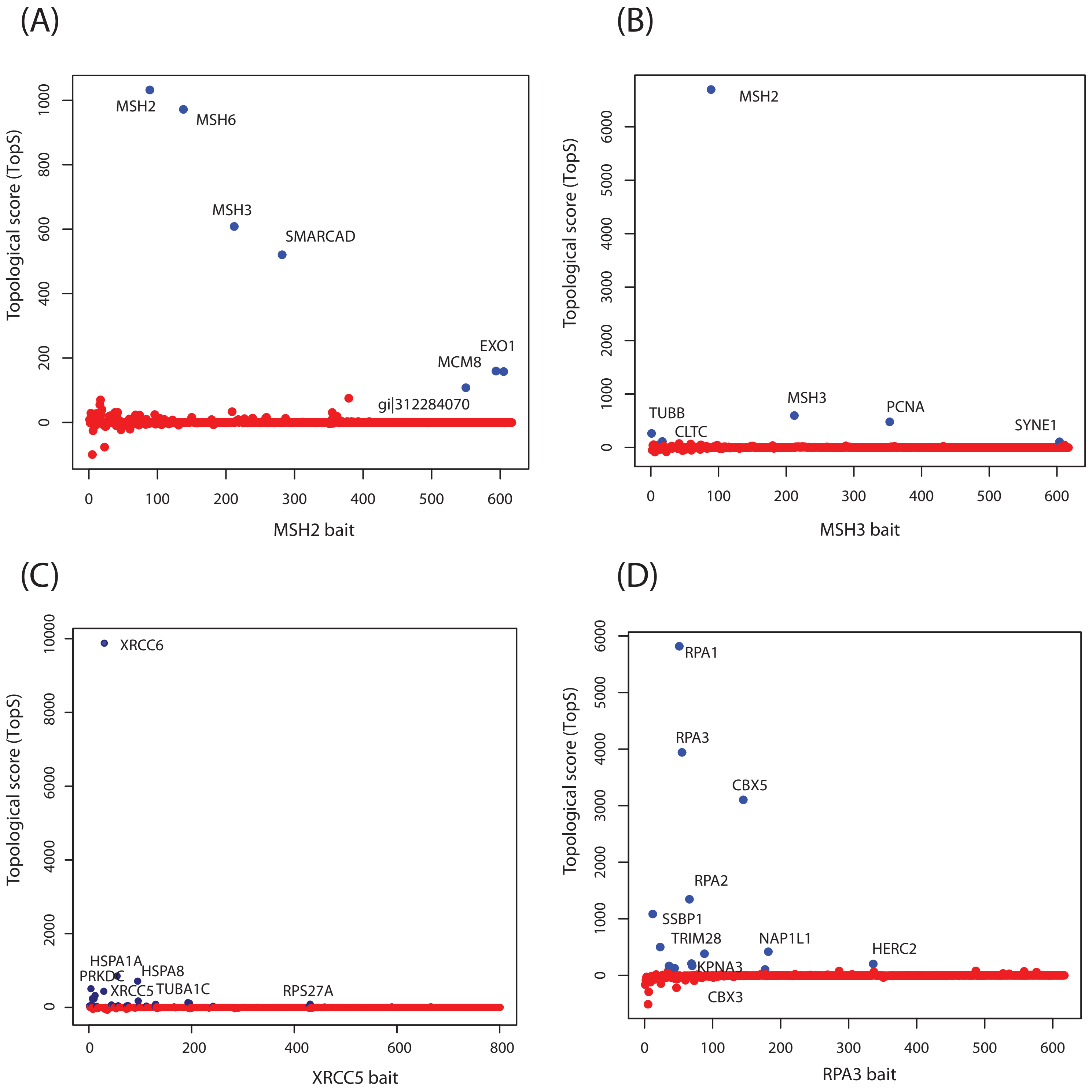


**Supplementary Figure 3. Distribution of the topological scores for proteins in baits involved in DNA repair.** Proteins with a TopS >100 are in blue and represent the strongest interactions in the MSH2 (A), MSH3 (B), XRCC6 (C) and RPA3 (D) baits.

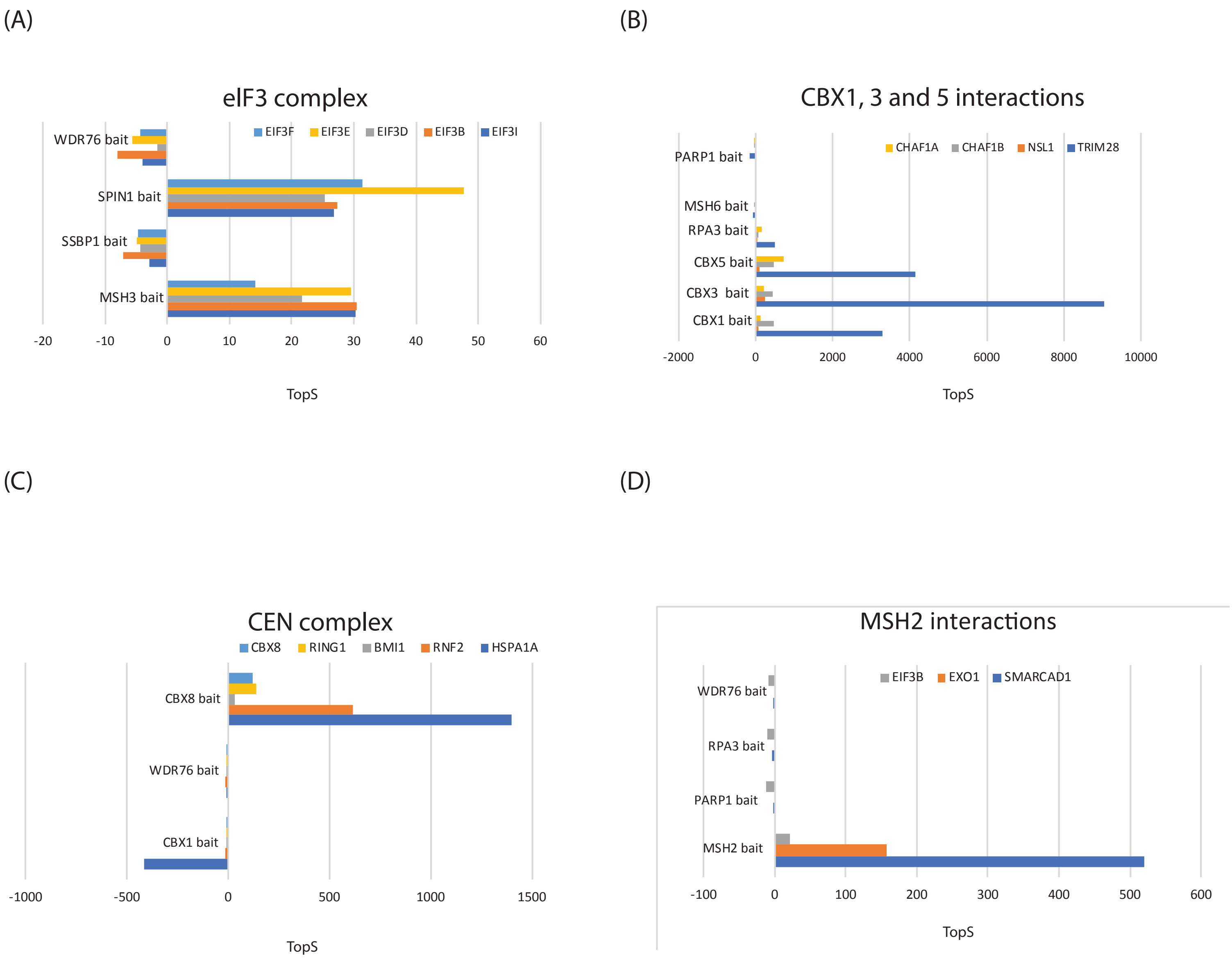


**Supplementary Figure 4. Representation of the TopS values for a selected number of complexes and biologically-related proteins.** (A) TopS values for the subunits of the eIF3 complex measured in WDR76, SPIN1, SSBP1 and MSH3 baits. (B) Four proteins (CHAF1A, CHAF1B, NSL1 and TRIM28) with high TopS values with the CBX1, 3 and 5 baits and negative scores in PARP1, MSH6 and RPA3 baits. (C) Five subunits of the CEN complex (CBX8, RING1, BMI1, RNF2 and HSPA1A) with high TopS values for the CBX8 bait and negative scores with WDR76 and CBX1 baits. (D) EF3B, EXO1, and SMARCAD1 with high TopS values for the MSH2 bait and negative scores in the WDR76, RPA3 and PARP1 APMS.

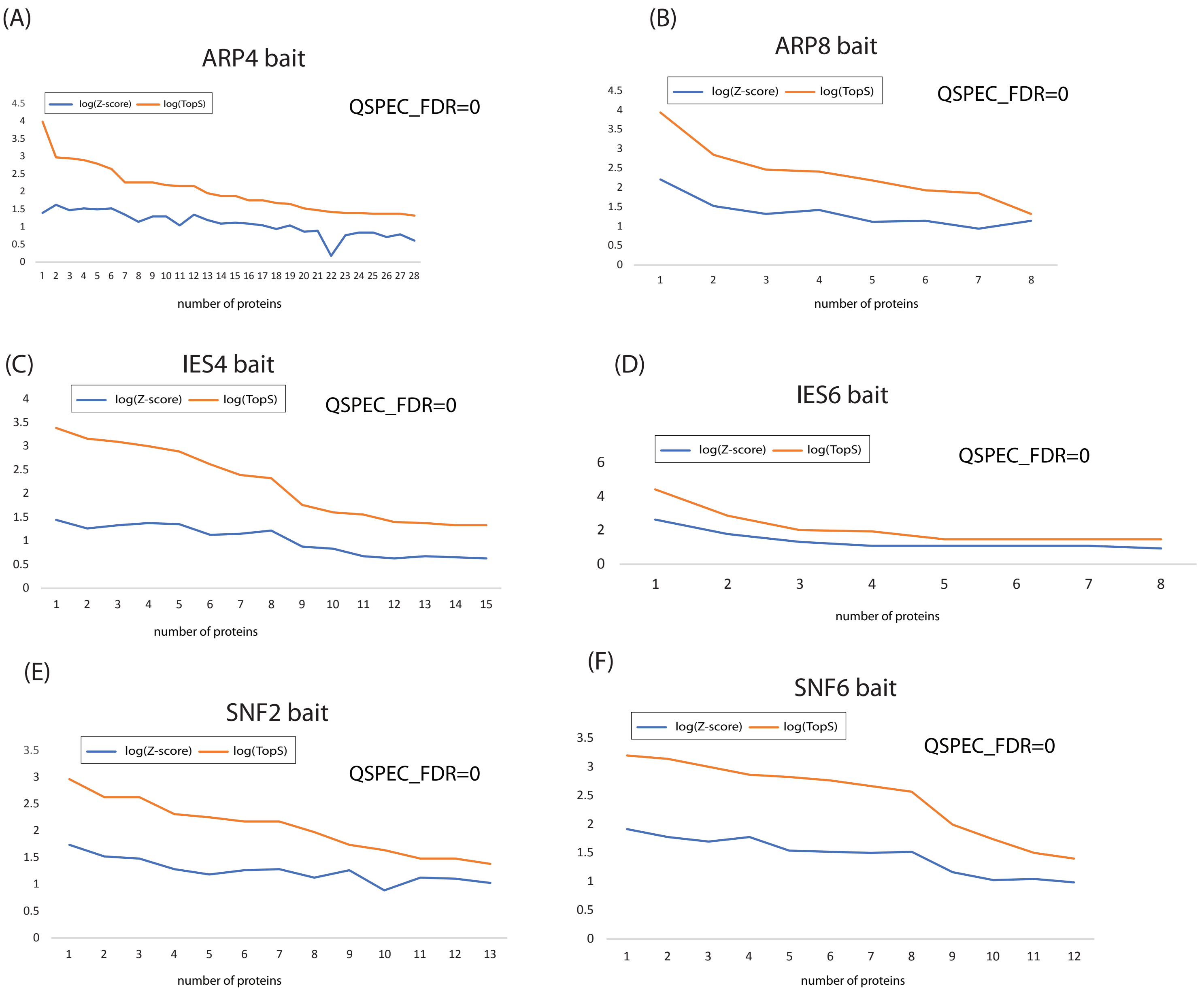


**Supplementary Figure 5. Distribution of the TopS, Z-scores and FDR values from QSPEC for the baits in the yeast datasets.** Number of proteins within a TopS threshold greater than 20 versus logarithm of TopS and Z-scores in the following baits: (A) ARP4, (B) ARP8, (C) IES4, (D) IES6, (E) SNF2 and (F) SNF6.

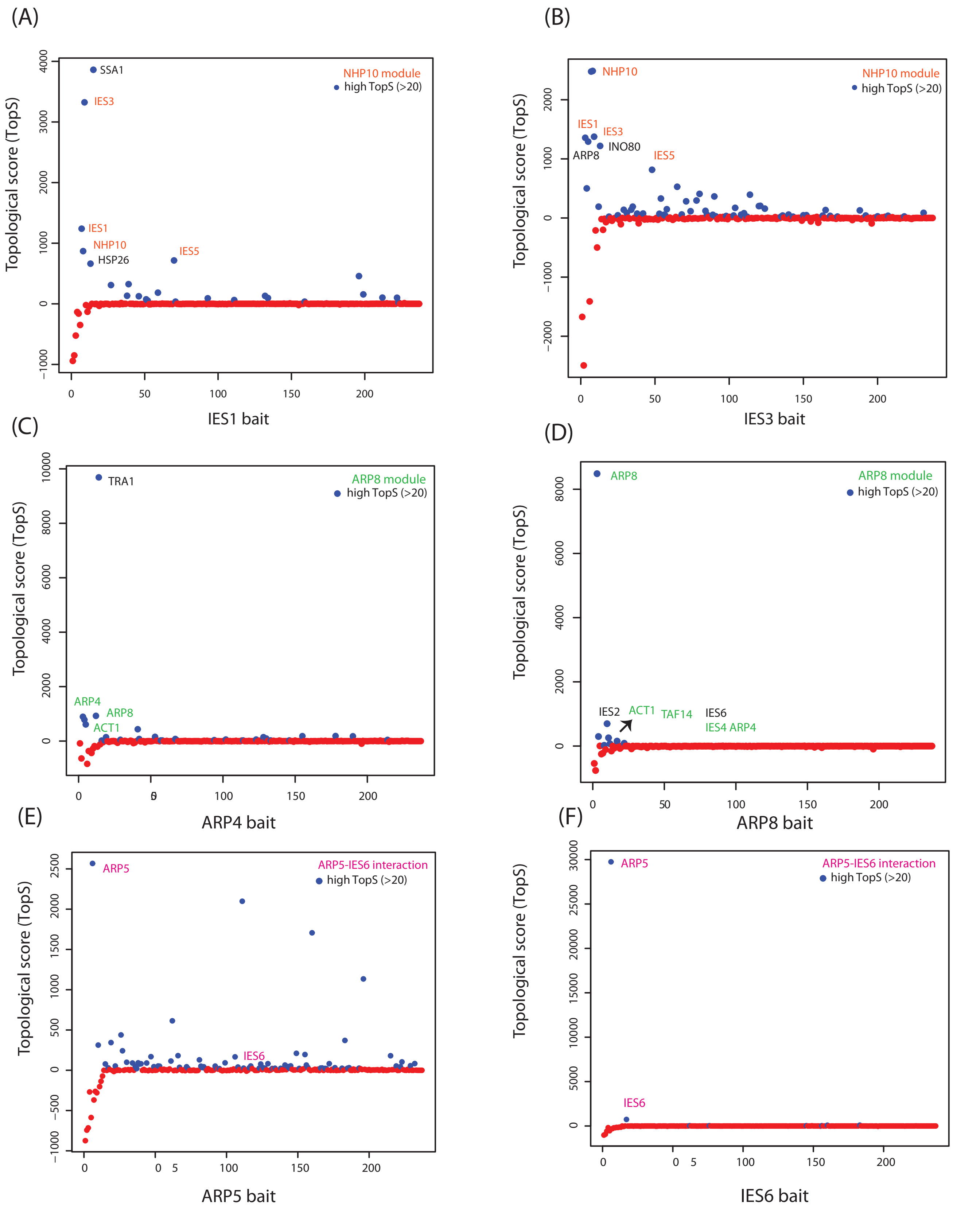


**Supplementary Figure 6. Distribution of the topological scores for proteins in baits belonging to different INO80 modules.** Proteins with a TopS >20 are in blue and represent the strongest interactions in the NHP10 module (A-B), the ARP8 module (C-D), and the ARP5-IES6 interaction (E-F).

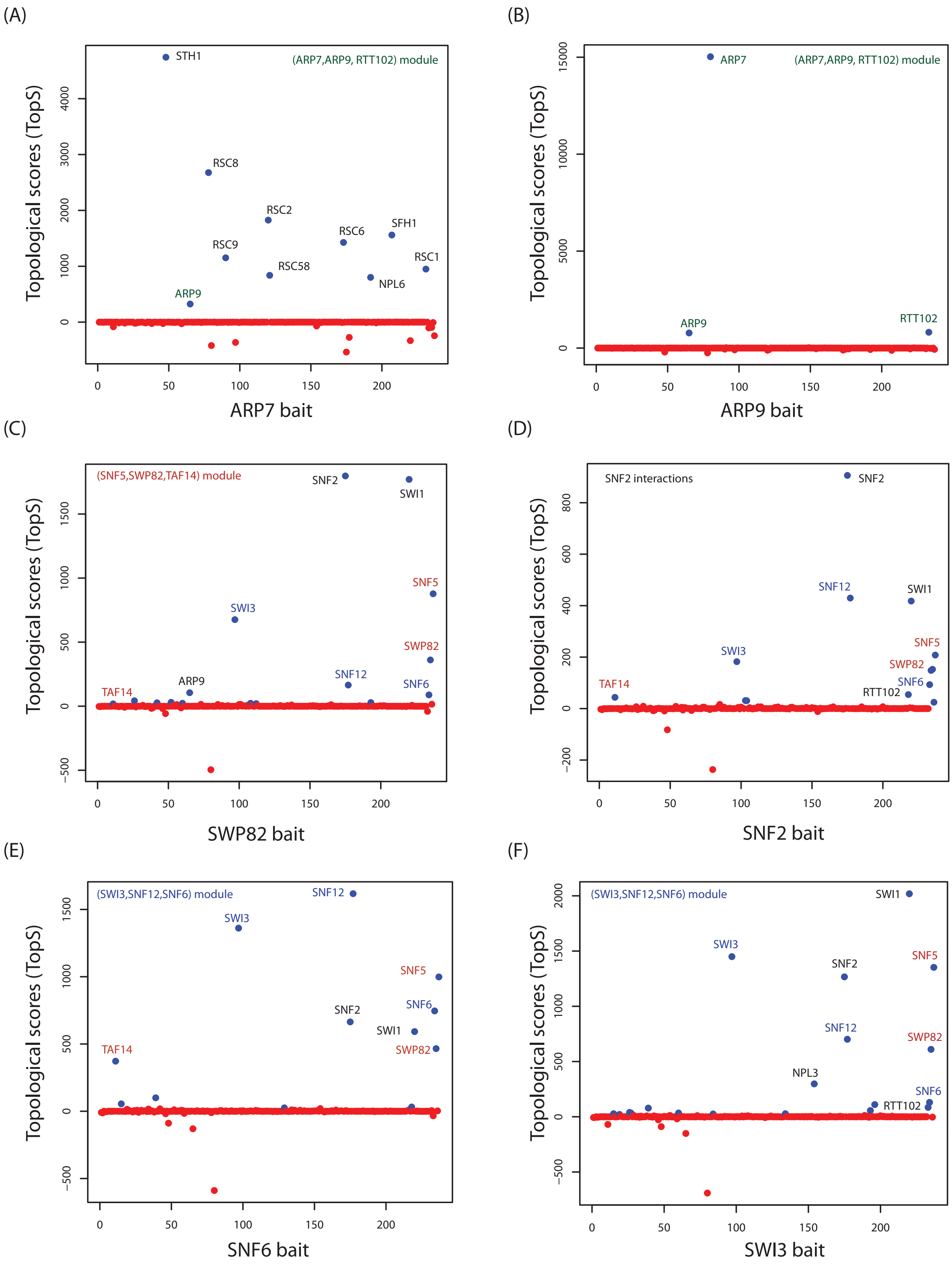


**Supplementary Figure 7. Distribution of the topological scores for proteins in baits belonging to different SWI/SNF modules.** Proteins with a TopS >20 are in blue and represent the strongest interactions in the (ARP7, ARP9 and RTT102) module (A-B), the (SNF5, SWP82 and TAF14) module (C), the SNF2 interactions (D), and the (SWI3, SNF12, and SNF6) module (E-F).

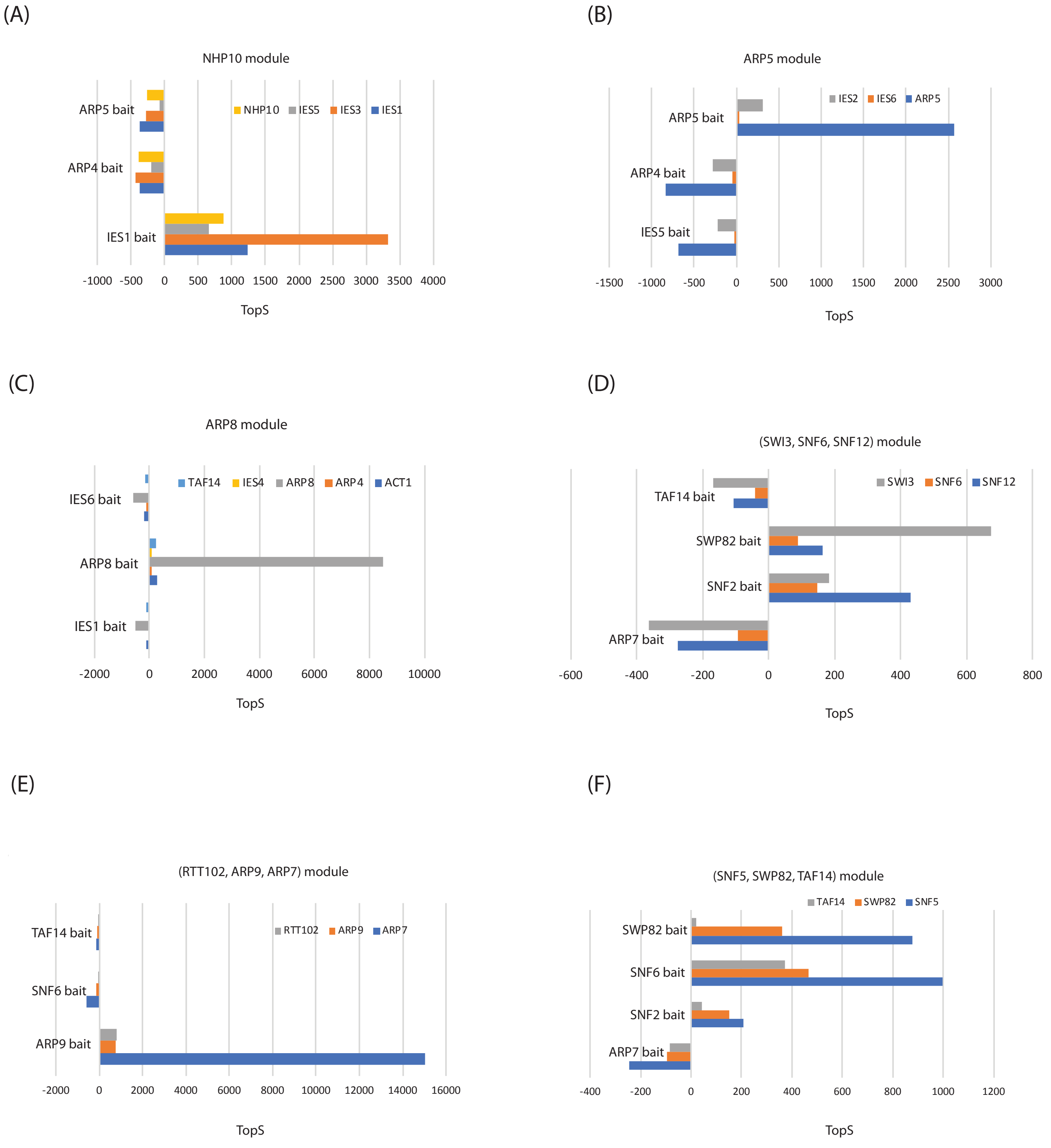


**Supplementary Figure 8. Representation of the TopS values for modules in the INO80 and SWI/SNF complexes.** (A) Four components of the NHP10 module show high TopS values with the IES1 bait and negative scores in ARP5 and ARP4 baits, which are subunits of different modules of the INO80 complex. (B) Three subunits of the ARP5 module show high TopS values with the ARP5 bait and negative scores with ARP4 and IES5 baits. (C) Five members of the ARP8 modules show high TopS values in ARP8 bait and negative scores in IES6 and IES1 baits, which are part of different modules within the INO80) complex. (D) Subunits of the (SWI3, SNF6 and SNF12) module display high scores with SWP82 and SNF2 baits and negative scores with ARP7 and TAF14 baits, which are subunits of different modules of the SWI/SNF complex. (E) Three proteins of the (ARP7, ARP9 and RTT102) module show high scores with the ARP9 bait as expected and negative scores with the TAF14 and SNF6 baits. (F) Three members of the (SNF5, SWP82 and TAF14) module have high TopS values for the SWP82, SNF6, and SNF2 baits and negative scores in the ARP7 bait.

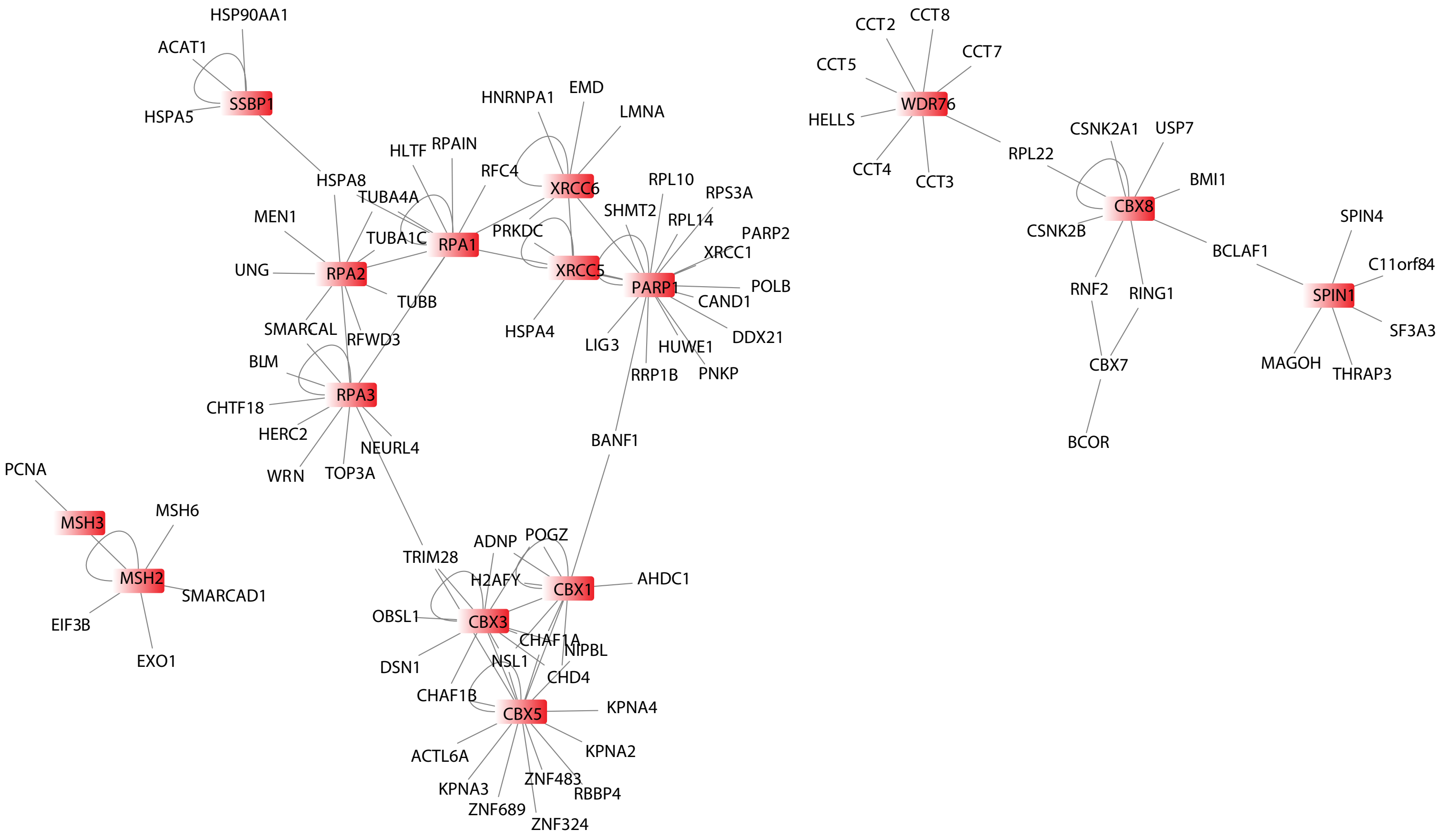


**Supplementary Figure 9. Representation of the protein interactions within the human DNA repair network with TopS values greater than 20 and interactions reported in the BioGRID database**. The baits in red and the prey proteins in black. A total of 130 proteins pairs (including 9 baits with themselves) are included in this network generated using Cytoscape.

(A)

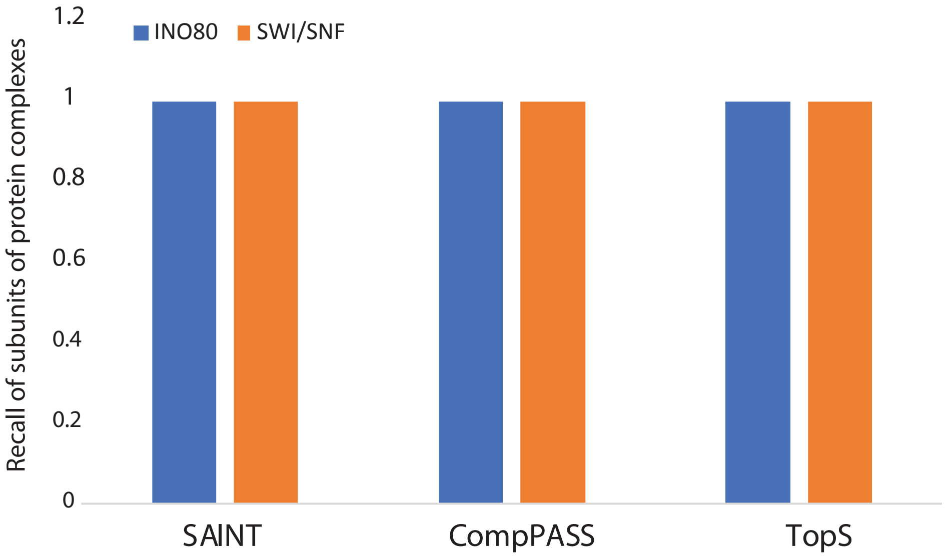


(B)

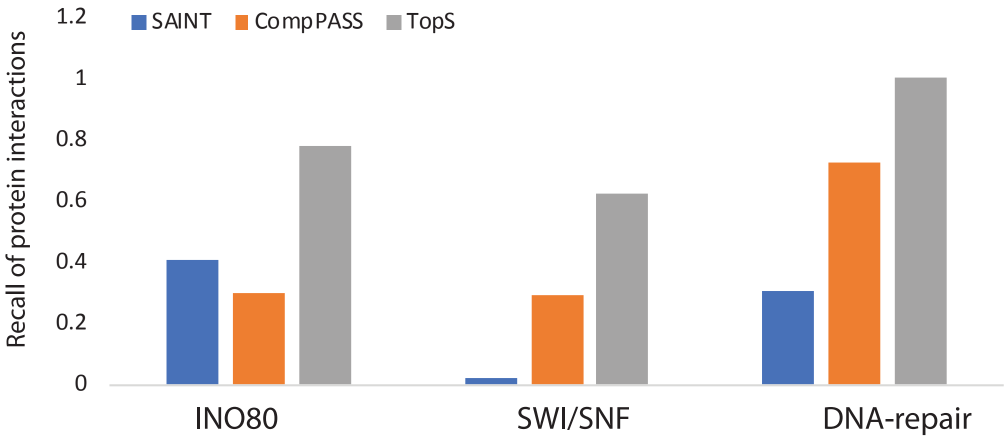


**Supplementary Figure 10. Comparison of SAINT, CompPASS, and TopS.**
(A) Comparison of the recall of the subunits of INO80 and SWI/SNF complexes using SAINT, CompPASS and TopS. Bait affinity purifications were compared to negative control purifications using standard approaches. The recall for the 15 subunits of the INO80 complex and 12 subunits for the SWI/SNF are plotted for all three methods. (B) Comparison of the recall of potential direct protein interactions in INO80, SWI/SNF and DNA repair datasets using SAINT, CompPASS, and TopS. The recalls for the 75 well-characterized protein interactions from crosslinking data for the INO80 and SWI/SNF datasets and 121 protein interactions from benchmarking against Bio-GRID for the DNA repair dataset are plotted.
