# Supplementary material for: Topological scoring of protein interaction networks
Source: Nat Commun. 2019 Mar 8;10:1118. doi: 10.1038/s41467-019-09123-y (PMC6408525; doi:10.1038/s41467-019-09123-y)
Supplement: Supplementary file 3 — Description of Additional Supplementary Files [file 41467_2019_9123_MOESM3_ESM.docx]

**Description of Additional Supplementary Files**

File Name: Supplementary Data 1

Description: **Human DNA Repair Dataset.** A. Proteins detected by MudPIT analyses of affinity purifications from 17 Halo-tagged proteins involved in DNA repair. For each protein are reported the number of detected peptides, the distributed spectral counts, sequence coverage, and dNSAF values. For each bait APMS are reported the dNSAF values averaged across three replicates, the frequency of detection, and the ratios of averaged bait dNSAF divided by control dNSAF. B. QSPEC analysis of each bait vs the Halo-AP control runs. Distributed spectral counts for each of the triplicate APMS analyses were inputted along with protein length and the corresponding spectral counts measured in the negative controls (S0). QSPEC computed LogFold-Change, Z statistics, and FDR values for all identified proteins. C. List of proteins significantly enriched in at least one bait compared to negative controls. A total of 801 proteins passed the z-score >=2 and FDR<0.01 criteria.

File Name: Supplementary Data 2

Description: **Scoring Interactions within the Human DNA Repair Dataset.** A. Bait Normalization. To adjust for bait enrichment, the distributed spectral counts of bait proteins in their APMS runs were replaced by the spectral counts values generated by Equation (1). B. Topological Scores. The TopS algorithm was used to derive topological scores for each of the 801 significant proteins in each of the 17 APMS analyses. C. Summary of TopS and QSPEC analyses of each bait vs all other baits. TopS values were computed from one TopS analysis across the matrix of 801 significant proteins x 17 APMS. Because each bait had to be compared against all other baits in the dataset, 17 QSPEC analyses were performed. D. List of proteins with a topological score >=20 in at least one bait.

File Name: Supplementary Data 3

Description: **Known Complexes within the Human DNA Repair Dataset**. A. Proteins with TopS greater than 20 sorted by complexes using the CORUM source. Over 600 proteins with a TopS value greater than 20 in at least one bait (Supplementary Data 1C) were inputted into the CORUM database of mammalian protein complexes. B. Bait/complexes relationships. Each of the 17 DNA repair baits were assigned to the various complexes of which they are known members. C. Shared patterns of TopS interaction confidence for complex members. TopS values greater than 2 were highlighted in red to illustrate that proteins belonging to the same complexes displayed bait-specific patterns of positive TopS. D. Scattered distribution of positive TopS values for proteins belonging to multiple complexes. This list reports 43 proteins whose positive TopS values did not show bait-specific patterns. These proteins were shared between multiple complexes. E. Divergent patterns of TopS values for proteins within the same complexes. This list reports 17 proteins whose positive TopS values did not comply with the consensus observed for other subunits of their known complex(es) (Supplementary Data 3C).

File Name: Supplementary Data 4

Description: **Topological Data Analysis of the Human DNA Repair Dataset.** Proteins with TopS >=20 were analyzed by TDA using their TopS values as input. Eight TDA clusters were identified.

File Name: Supplementary Data 5

Description: **Analysis of the Drug-Gene Enrichment within the Human DNA Repair Dataset.** The enrichment was determined for proteins with a TopS ≥20 in at least one bait. Drug-gene enrichment was generated from the WebGestalt database. Nine classes were identified with significant p-values. Statistical parameters are reported for each of the nine enriched classes.

File Name: Supplementary Data 6

Description: **Yeast INO80 Complex Dataset.** A. Proteins detected by MudPIT analyses of affinity purifications from 14 TAP-tagged subunits of the *Saccharomyces cerevisiae* INO80 complex. For each protein are reported the number of detected peptides, spectral counts, unique spectral counts, uniquely detected peptides, shared spectral counts, distributed spectral counts, sequence coverage, and dNSAF values. B. Contaminant Extraction. The non-specific proteins were extracted based on the ratio between affinity purifications and TAP negative controls. For each bait APMS are reported the dS values averaged across replicates, when applicable. C. Intersection between the INO80 and SWI/SNF datasets. 237 proteins significantly enriched in the INO80 dataset were shared with the SWI/SNF dataset (Supplementary Data 7A). D. Input matrix for TopS analysis. Distributed spectral counts for the NHP10 bait were normalized using Equation (1) as described in the main text. E. TopS results for the 237 proteins significant in the INO80 and SWI/SNF datasets. Protein pairs with TopS greater than 20 are in bold. F. TopS results. Same as in (E) but the interactions are represented in a different format. G. Comparison of topological scores with protein crosslinking results. The reported crosslinking interactions agreed with the TopS values. Interactions with a positive TopS are represented in red.

File Name: Supplementary Data 7

Description: **Yeast SWI/SNF Complex Dataset.** A. Proteins detected by MudPIT analyses of affinity purifications from 10 TAP-tagged subunits of the *Saccharomyces cerevisiae* SWI/SNF complex. For each protein are reported the number of detected peptides, spectral counts, unique spectral counts, uniquely detected peptides, shared spectral counts, distributed spectral counts, sequence coverage, and dNSAF values. For each bait APMS are reported the dNSAF values averaged across three replicates, the frequency of detection, and the ratios of averaged bait dNSAF divided by control dNSAF. B. Intersection between the INO80 and SWI/SNF datasets. 237 proteins significantly enriched in the SWI/SNF dataset were shared with the ION80 dataset (Supplementary Data 6A). C. Input matrix for TopS analysis. Distributed spectral counts for the ARP9 bait were normalized using Equation (1) as described in the main text. D. TopS results for the 237 proteins significant in the INO80 and SWI/SNF datasets. Protein pairs with TopS greater than 20 are in bold. E. TopS results. Same as in (D) but the interactions are represented in a different format. F. TopS values for all 237 proteins significantly enriched in both complexes. G. Comparison of topological scores with protein crosslinking results. The reported crosslinking interactions ^6^ agreed with the TopS values. Interactions with a positive TopS are represented in red.

File Name: Supplementary Data 8

Description: **Alternative Pipeline Analyses of the Human DNA repair Dataset.** A. QSPEC results. Every bait was compared against all other baits in the dataset. Seventeen QSPEC analyses were performed on 801 DNA repair proteins. B. SAINT results. Seventeen SAINT comparisons were performed as in (A). C. CompPASS results. CompPASS computation was performed on the averaged spectral counts. D. Intersection between TopS and CompPASS. CompPASS Z and WD scores were used for the comparison with TopS values. The two scoring methods were in agreement for 787 protein pairs.

File Name: Supplementary Data 9

Description: **Alternative Pipeline Analyses of the *S. cerevisiae* INO80 complex.** A. QSPEC results. Every bait was compared against all other baits in the dataset. Fourteen QSPEC analyses were performed on 237 proteins significantly enriched in the INO80 and SWI/SNF datasets. B. SAINT results. Fourteen SAINT comparisons were performed as in (A). C. CompPASS results. CompPASS computation was performed on the averaged spectral counts. D. Intersection between CompPASS and TopS. CompPASS Z and WD scores were used for the comparison with TopS values. 123 protein interactions passed selection criteria for both methods. E. Comparison of interaction confidence scores with protein crosslinking results. Most of the reported crosslinking interactions reported within the INO80 complex ^5^ agreed with at least two of the four scores. Interactions scores passing threshold are represented in red. F. INO80 subunits. TopS, QSPEC, CompPASS and SAINT results for the 15 subunits of the INO80 complex.

File Name: Supplementary Data 10

Description: **Alternative Pipeline Analyses of the *S. cerevisiae* SWI/SNF complex.** A. QSPEC results. Every bait was compared against all other baits in the dataset. Ten QSPEC analyses were performed on 237 proteins significantly enriched in the INO80 and SWI/SNF datasets. B. SAINT results. Ten SAINT comparisons were performed as in (A). C. CompPASS results. CompPASS computation was performed on the averaged spectral counts. D. Comparison of interaction confidence scores with protein crosslinking results. Most of the reported crosslinking interactions reported within the SWI/SNF complex agreed with at least two of the four scores. Interactions scores passing threshold are represented in red. E. SWI/SNF subunits. TopS, QSPEC, CompPASS and SAINT results for the 15 subunits of the INO80 complex.

File Name: Supplementary Data 11

Description: **Polycomb Complexome Dataset**. A. Published peptide counts, spectral counts, and scores. WD^N^ scores from the Polycomb complexome dataset were used for the comparison with TopS analysis. B. TopS Analysis. TopS values were computed on 9853 candidate interactions and 64 baits from the polycomb complexome dataset. C. Intersection between TopS and CompPASS. At a TopS threshold of 2 and WD^N^ greater than 1.5, 1183 interactions overlapped. D. Comparison with BioGRID. 121 protein interactions (excluding 9 baits with themselves) were found in the intersection of BioGRID ^7^ and protein interactions with TopS values of 20 or higher.
