# Supplementary material for: Topological scoring of protein interaction networks
Source: Nat Commun. 2019 Mar 8;10:1118. doi: 10.1038/s41467-019-09123-y (PMC6408525; doi:10.1038/s41467-019-09123-y)
Supplement: Supplementary file 15 — Reporting Summary [file 41467_2019_9123_MOESM15_ESM.pdf]

nature research

Corresponding author(s): *Double-blind peer review submissions: write DBPR and your manuscript number here instead of author names.*

Reporting Summary

Nature Research wishes to improve the reproducibility of the work that we publish. This form provides structure for consistency and transparency in reporting. For further information on Nature Research policies, see Authors & Referees and the Editorial Policy Checklist.

Statistical parameters

When statistical analyses are reported, confirm that the following items are present in the relevant location (e.g. figure legend, table legend, main text, or Methods section).

n/a	Confirmed	
☐	☒	The exact sample size (*n*) for each experimental group/condition, given as a discrete number and unit of measurement
☐	☒	An indication of whether measurements were taken from distinct samples or whether the same sample was measured repeatedly
☒	☐	The statistical test(s) used AND whether they are one- or two-sided<br>*Only common tests should be described solely by name; describe more complex techniques in the Methods section.*
☒	☐	A description of all covariates tested
☒	☐	A description of any assumptions or corrections, such as tests of normality and adjustment for multiple comparisons
☒	☐	A full description of the statistics including central tendency (e.g. means) or other basic estimates (e.g. regression coefficient) AND variation (e.g. standard deviation) or associated estimates of uncertainty (e.g. confidence intervals)
☐	☒	For null hypothesis testing, the test statistic (e.g. *F*, *t*, *r*) with confidence intervals, effect sizes, degrees of freedom and *P* value noted<br>*Give P values as exact values whenever suitable.*
☒	☐	For Bayesian analysis, information on the choice of priors and Markov chain Monte Carlo settings
☒	☐	For hierarchical and complex designs, identification of the appropriate level for tests and full reporting of outcomes
☒	☐	Estimates of effect sizes (e.g. Cohen's *d*, Pearson's *r*), indicating how they were calculated
☐	☒	Clearly defined error bars<br>*State explicitly what error bars represent (e.g. SD, SE, CI)*

Our web collection on statistics for biologists may be useful.

Software and code

Policy information about availability of computer code

Data collection	TopS is written using Shiny application (R package version 3.4.2) for R statistics software. TopS uses several packages, including gplots, devtools and gridExtra. TopS is freely available at https://github.com/WashburnLab/Topological-score-TopS-.
Data analysis	Software used in this manuscript: Qspec version 1.2.2; SAINTexpress-v3.6.3-2018; CompPASS; ClustVis for clustering (Fig. 2A); PermutMatrix (Fig.3C, Fig.6B and Fig7C); Ayasdi platform (AYASDI Inc., Menlo Park CA) (Fig. 3A,Fig.6A, and Fig.7A), WebGestalt 2013 (Fug.7A).

For manuscripts utilizing custom algorithms or software that are central to the research but not yet described in published literature, software must be made available to editors/reviewers upon request. We strongly encourage code deposition in a community repository (e.g. GitHub). See the Nature Research guidelines for submitting code & software for further information.

April 2018

Data

Policy information about availability of data

All manuscripts must include a data availability statement. This statement should provide the following information, where applicable:

- Accession codes, unique identifiers, or web links for publicly available datasets
- A list of figures that have associated raw data
- A description of any restrictions on data availability

All the DNA repair data used here are deposited at the https://massive.ucsd.edu/ with MassIVE ID # MSV000081377. The data used for the analysis of the S. cerevisiae INO80 and SWI/SNF chromatin remodeling complexes are from previous studies on INO80 (MSV000079138) and SWI/SNF (MSV000081417).
Figures associated with this data: Fig.2, Fig. 3, Fig. 4,Fig.5,Fig.6, Supplementary Figs. 1-8, Supplementary Fig.10
Available datasets: polycomb dataset at: https://www.sciencedirect.com/science/article/pii/S2211124716312098

Field-specific reporting

Please select the best fit for your research. If you are not sure, read the appropriate sections before making your selection.

☒ Life sciences ☐ Behavioural & social sciences ☐ Ecological, evolutionary & environmental sciences

For a reference copy of the document with all sections, see nature.com/authors/policies/ReportingSummary-flat.pdf

Life sciences study design

All studies must disclose on these points even when the disclosure is negative.

Sample size	not applicable
Data exclusions	not applicable
Replication	not applicable
Randomization	not applicable
Blinding	not applicable

Reporting for specific materials, systems and methods

Materials & experimental systems

n/a	Involved in the study	
☐	☒	Unique biological materials
☒	☐	Antibodies
☐	☒	Eukaryotic cell lines
☒	☐	Palaeontology
☒	☐	Animals and other organisms
☒	☐	Human research participants

Methods

n/a	Involved in the study	
☒	☐	ChIP-seq
☒	☐	Flow cytometry
☒	☐	MRI-based neuroimaging

Unique biological materials

Policy information about availability of materials

Obtaining unique materials	All materials are freely available.

Eukaryotic cell lines

Policy information about cell lines

Cell line source(s)	Flip-In HEK293T cells were purchased from Thermo Fisher Scientific (Waltham, MA).

Authentication	Cells were authenticated using the ATCC (Manassas, VA) cell line authentication service.
Mycoplasma contamination	Cells are tested quarterly for mycoplasma and using the ATCC Mycoplasma detection kit (#30-1012K).
Commonly misidentified lines (See ICLAC register)	*Name any commonly misidentified cell lines used in the study and provide a rationale for their use.*
